# The transcultural adaptation and validation of the Chinese version of the Attitudes Toward Recognizing Early and Noticeable Deterioration scale

**DOI:** 10.3389/fpsyg.2022.1062949

**Published:** 2022-12-06

**Authors:** Wenbo Li, Hongyu Yu, Bing Li, Yanli Zhang, Mingshu Fu

**Affiliations:** ^1^Department of Nursing, Jinzhou Medical University, Jinzhou, China; ^2^Department of Dermatology, Shengjing Hospital of China Medical University, Shenyang, China; ^3^Department of Neurosurgery, The First Affiliated Hospital of China Medical University, Shenyang, China

**Keywords:** attitudes, clinical deterioration, ward nurses, patient assessment, reliability, validity

## Abstract

**Background:**

In China, clinical deterioration events present a real problem for every clinical nurse. Patient deterioration is determined in part by nurses’ attitudes toward early recognition of clinical deterioration. However, research on attitudes toward the early identification of clinical deterioration is still in its infancy, and even less research has been done on ward nurses’ attitudes toward the early identification of clinical deterioration. To drive behavioral change and improve the care of deteriorating patients, nurses need comprehensive, valid, and reliable tools to assess their attitudes toward early identification of deterioration.

**Objective:**

In this study, we aimed to translate the Attitudes Toward Recognizing Early and Noticeable Deterioration (ATREND) scale into Chinese and to assess its validity and reliability tests.

**Methods:**

From March 2022 to July 2022, the ATREND scale was translated, back-translated, and cross-culturally adapted into the Chinese version using a modified Brislin translation model. Then, 460 ward nurses were recruited from tertiary Grade A general hospitals in two cities: Shenyang and Jinzhou in Liaoning Province, China. Reliability analyses were conducted using internal consistency, split-half, and test–retest reliability. We convened a committee of experts to determine the validity of the content. Tests of the structural validity of the scale were conducted using exploratory and validation factor analyses.

**Results:**

The Cronbach’s α value of the Chinese version of the ATREND scale was 0.804, and the Cronbach’s α value of the dimensions ranged from 0.782 to 0.863. The split-half reliability and test–retest reliability were 0.846 and 0.711, respectively. Furthermore, the scale has an index of content validity of 0.922, indicating a high level of content validity. In exploratory factor analysis, eigenvalues, total variance explained, and scree plot supported a three-factor structure. The three-factor model supported by this study was confirmed by confirmatory factor analysis (CFA). Moreover, the model fitting indexes (e.g., *χ*^2^/DF = 1.498, GFI = 0.954, RMSEA = 0.047) were all within acceptable limits based on the CFA.

**Conclusion:**

The Chinese version of the scale is reliable and valid among ward nurses. Nursing educators and clinicians will be able to develop targeted educational programs to enhance the competence and behaviors of Chinese ward nurses in recognizing clinical deterioration. It will be based on the developed scale to assess Chinese nurses’ attitudes and practices regarding early recognition of clinical deterioration. As a result, it is necessary to consider the Chinese scale’s three-factor structure. The developed three-factor structured scale will assess Chinese ward nurses’ attitudes and practices toward patient observation and vital sign-monitoring empowerment, enlightening them on the importance of patient observation, encouraging ward nurses to use a wider range of patient assessment techniques to capture early signs of clinical deterioration, and helping ward nurses to develop clinical confidence to monitor clinical deterioration.

## Introduction

As defined by the medical profession, clinical deterioration refers to “a change from a clinically healthy state to an increasingly poor health state that increases the risk of death, disability, organ dysfunction, or illness (Jones et al., 2013; Treacy et al., 2022).” The rapid response system (RRS) is a mechanism for the early identification of critically ill patients at potential risk of adverse events using single or multiple early warning values in the setting of a continuing shortage of critical care resources and equipment and then accurately initiating rapid response team, medica emergency teams, or critical care outreach team to provide the targeted response and acute management of ward patients (McGaughey et al., 2017; Robertson et al., 2021). The implementation of RRSs has gained increasing attention over the past two decades to identify and respond promptly to deteriorating inpatients as part of the global patient quality and safety agenda (Winters et al., 2013; McGaughey et al., 2021). A nurse’s role in recognizing and responding to clinical deterioration is crucial as they are often the first witnesses to changes in a patient’s condition (Mohammmed Iddrisu et al., 2018; Chua et al., 2019). Nurses’ ability to recognize, document, and report changes in vital signs can significantly impact the regression of changes in patients’ conditions (Mohammmed Iddrisu et al., 2018). Despite the introduction of essential sign-monitoring tools that incorporate early warning scoring systems in hospitals, ward nurses still have difficulty identifying and treating acute clinical deterioration (Liaw et al., 2016; McKinney et al., 2019; Gerry et al., 2020). It is possible to increase adverse events such as morbidity and mortality, unplanned transfers to the intensive care unit (ICU), and cardiopulmonary arrest if nurses fail to monitor and report changes in vital signs (Egilsdottir et al., 2021; Smith et al., 2022). For this reason, it is essential for the patient’s health that they maintain vigilance and respond appropriately to any deterioration in their condition.

Hospitalized patients who deteriorate in the general ward setting and are not recognized as deteriorating are at risk for clinical deterioration, including unexpected admission to the ICU, respiratory or cardiac arrest, or death (Smith et al., 2022). Studies have shown that approximately 50% of serious adverse events are preventable (Ko et al., 2020). Physiological deterioration often precedes the most serious adverse events, usually occurring within a few minutes to 24 h (Al-Moteri et al., 2019). It has been shown that 70% of hospitalized patients experience severe vital sign changes 6–8 h before cardiac arrest, with abnormal breathing being the most common (Mochizuki et al., 2017; Wu et al., 2021). It has led to the widespread implementation of hospital-based vital sign tracking and early warning devices (Brekke et al., 2019). By using the physiological tracking and triggering tool, healthcare professionals will be able to identify patients at risk for clinical deterioration and initiate interventions to prevent further clinical deterioration based on escalation protocols (Pullyblank et al., 2020). When a nurse receives an alert, they must “trigger” an escalation process that specifies clinical actions and time frames for responses (Petersen et al., 2017). Even with physiological tracking and trigger tools in hospitals, patient deterioration often remains undetected or missed, resulting in adverse outcomes (Credland et al., 2018). Several studies have shown that infrequent and incomplete vital sign measurements and charting (Redfern et al., 2019), insufficient knowledge of normal vital sign values (Difonzo, 2019), inadequate knowledge of some vital signs (Difonzo, 2019), inadequate supervision of nursing staff performing vital sign monitoring (Downey et al., 2022), excessive workload (Dall'Ora et al., 2021), and prioritization of vital sign readings over clinical judgment (Massey et al., 2017) are several risk factors that contribute to the failure of ward nurses to identify deteriorating patients. It was found that as the operation of vital signs was relatively simple and vital signs became a daily task in nursing, nurses did not pay enough attention to them, learned less about them, and even failed to record the values of vital signs truthfully, such as writing respiratory rate as a normal range value just by normal oxygen saturation (Weenk et al., 2019; Nicolò et al., 2020). Second, inadequate supervision by nursing managers can lead to inadequate nursing staff commitment and affect the standardization of clinical practice (Manning, 2016), leading to failure to promptly recognize the onset of early deterioration. It has also been suggested that it is one of the main influences on patient safety outcomes (Squires et al., 2010; Ying et al., 2021). In addition, insufficient nursing human resources is a problem in both domestic and foreign medical institutions, especially when the number of patients increases or when there are more acute and critically ill patients, the nursing workload is great, and nurses focus on doing the main nursing operations with insufficient time and neglect the basic condition assessment (Ball et al., 2018; Dall'Ora et al., 2019). Studies have also found that inexperienced nurses rely on machines and equipment and lack an active thinking process that facilitates overall patient assessment, which hinders and delays the recognition of deterioration (Massey et al., 2017). It has been reported that as experience increases, nurses’ clinical judgment and decision-making skills improve accordingly, and good clinical judgment enables nurses to detect changes in patients’ conditions promptly so that early intervention can be given (Redfern et al., 2019; Oh et al., 2022). Nursing managers can develop prevention strategies for these potentially modifiable factors, representing a potential starting point for promoting early recognition of clinical deterioration by ward nurses. Patients also show early subtle abnormal changes in their clinical condition, and the importance of some signs and symptoms as early signs of deterioration has been confirmed in several studies, such as respiratory changes, circulatory changes, mental status changes, cognitive changes, behavioral changes, and acute pain (Douw et al., 2015; Khanna et al., 2019). Patients may experience symptoms such as shortness of breath (dyspnea), night sweats, weakness, and chest pain before cardiac arrest (AC) (Bangaoil et al., 2020; Silva et al., 2022). Antonio Gangemi et al. found that cognitive decline was associated with significant changes in cognitive ability at pre-morbid baseline levels, among which patients with gastric ulcers and epilepsy were more likely to show early clinical signs of cognitive decline (Gangemi et al., 2021). Some of the symptomatic changes that occurred were subtle, such as the appearance of pale skin and agitated conditions (Cioffi et al., 2009). Identifying subtle and early clues to clinical deterioration and making clinical decisions is a complex cognitive and behavioral strategy (Evans et al., 2015), and along with routine vital sign measurements, it is necessary to keep a close eye on the patient throughout their hospitalization in order to detect subtle clues and provide an opportunity for early intervention. Clinical nurses spend the most time at the patient’s bedside and are often the first to observe and identify a patient’s condition (Brooks Carthon et al., 2019). The clinical nurse’s identification, documentation, and reporting of changes in a patient’s condition are important for patient transition (Liu W. et al., 2021). Therefore, effectively assessing ward nurses’ attitudes toward recognizing changes in patients’ conditions, taking effective measures, and providing targeted training to improve nurses’ alertness to changes in conditions are important to ensure patient safety and improve service quality.

In recent years, there has been increasing recognition of the need to address gaps in nurses’ actions for early identification and clinical deterioration (Smith et al., 2019; Walker et al., 2021). Despite gaining knowledge through health education, it is not generally enough to change behavior in a lasting way (Prochaska and Velicer, 1997). The theory of reasoned action asserts that an individual’s behavioral intentions are influenced by their attitudes, and intentions are the best predictors of their behavior (Fishbein and Ajzen, 1977). As an extension of the theory of reasoned action, the idea of planned behavior (TPB) suggests that attitudes play an essential role in decision-making (Dosman et al., 2001; Shen et al., 2020; Seong and Hong, 2021).

Generally, attitudes are beliefs, feelings, and behaviors related to events, people, and things (Taghva et al., 2022). Rather than being innate, it is formed through learning (Albarracín et al., 2014; Shim et al., 2016; Cao et al., 2022). Individuals’ attitudes reinforce their intentions and change their behavior, making reliable predictions about their likelihood of performing specific behaviors (Medisauskaite et al., 2021; Liu K. S. N et al., 2021). An interview with ward nurses who experienced resuscitation shows that nurses’ attitudes toward identifying early and significant deterioration seriously affected their ability to recognize, interpret, and report changes in conditions (Shearer et al., 2012; Chua et al., 2013). In addition, this attitude is associated with nurses’ perception, understanding, and prediction of clinical deterioration, workload, and trust and cooperation (Shearer et al., 2012; Mok et al., 2015b). In the intervention study (Saab et al., 2017), nurses who were more alert to clinical deterioration experienced fewer clinical adverse events. In addition, ward nurses’ attitudes toward early identification of clinical deterioration influence their compliance with follow-up and trigger tools, their vigilance toward patients at risk, and their perception of the importance of early identification of clinical deterioration in reducing adverse events (Wood et al., 2019). Nurses with positive attitudes are more attentive to changes in patients’ conditions, relate them to pathological changes, and respond early and proactively through effective healthcare communication, which has a crucial impact on improving the prognosis of patients’ diseases (Padilla and Mayo, 2018). Therefore, practical assessment of ward nurses’ attitudes regarding vital sign monitoring for identifying changes in patients’ conditions and targeted training on an evidence-based basis to improve nurses’ alertness to deterioration in vital signs are essential to ensure the safety of care and improve the quality of service (Kamio et al., 2018).

The attitude of ward nurses toward early recognition of clinical deterioration is essential for identifying clinical deterioration and preventing morbidity and mortality. However, it remains a significant challenge for nurses to detect these early signs of deterioration (Griffiths et al., 2018). To identify early and significant deterioration in wards, Singaporean scholars developed the Attitudes Toward Recognizing Early and Noticeable Deterioration (ATREND) scale in 2022 (Chua et al., 2022). It was mainly used to evaluate nurses’ attitudes toward recognizing early and significant deterioration with high reliability and validity. Currently, most research has focused on the perceptions and responses of clinicians and hospital administrators to the clinical deterioration of patients (Allen et al., 2018; Xiong et al., 2022). There is no quantitative assessment of nurses’ attitudes toward identifying early and significant ward deterioration in China. Having valid and reliable tools related to patient care may help improve the quality of health care. Nursing students and ward nurses can better identify clinical deterioration by identifying their attitudes toward early signs of deterioration, as Wei Ling Chua suggests (Chua et al., 2022). Therefore, this study aimed to introduce the English version of the ATREND scale into China through translation and cultural adaptation and to determine its reliability and validity.

## Materials and methods

### Design and participants

This cross-sectional and observational study aimed to translate the ATREND scale and test its reliability and validity in a Chinese version. This study was carried out in tertiary Grade A general hospitals in two cities, Shenyang and Jinzhou, Liaoning Province—from March 2022 to July 2022. We included participants if they (1) were active registered nurses, (2) had been engaged in clinical nursing in the ward for more than 6 months, and (3) volunteered for this study. A list of exclusion criteria is provided below: (1) Nurses engaged in intensive care and emergency care; (2) Nurses in training and internships; and (3) Nurses engaged in non-clinical nursing work such as logistics or research. Eventually, 460 clinical nurses were recruited through convenience sampling from hospitals with the help of nursing directors. We collected basic information about the socio-demographic characteristics of the participants (including age, gender, education level, work experience, job title, and department).

### Translation, back-translation, and transcultural adaptation of the ATREND scale

Permission to translate and adapt the ATREND scale was obtained from Dr. Wei Ling Chua, the author of the original version, by email. The English version of the ATREND scale was translated into a first draft of the Chinese version based on an adapted Brislin translation model, which included translation, back-translation, transcultural adaptation, and a pilot study (Brislin, 1970; Jones et al., 2001).

### Translation and back-translation

#### Step 1: Forward translation

Two native Chinese speakers fluent in English independently translated the English version of the scale to form the first Chinese version of the ATREND scale. Translator 1, a nursing graduate student who had passed the CET-6 and had foreign exchange experience, developed a clinically appropriate translation, ensuring equivalence between it and the original one. To reflect the language habits of the general public, translator 2, a master’s degree student in English without a medical background, translated from a linguistic perspective.

#### Step 2: Integration

It was decided to perform a comparative analysis of the two translations by another graduate nursing student who has a native Chinese-speaking background, is fluent in English, and was not involved in forwarding the translation. After discussing the differences among the three researchers, the second Chinese version of the ATREND scale was developed. The integration of differences in this stage was generated by consensus among the three researchers.

#### Step 3: Back-translation

Two nursing researchers with Ph.D. degrees translated the Chinese version into the back-translated version. Their knowledge of medical English was excellent, but neither had ever seen the original ATREND scale. The final step was to invite two other bilingual speakers to compare and adjust the two translated versions. The back-translators repeated the translation several times until the translation matched the original English version to form the Chinese version of the ATREND scale. The draft of the Chinese version of the ATREND scale was finally developed.

### Transcultural adaptation

#### Step 1: Expert consultation

We have invited some experts to modify the scale items to be more compatible with Chinese expressions and habits. An expert committee, including two professors from the Nursing College of Jinzhou Medical University, one associate chief nurse, two nurse practitioners charged with clinical work for more than 10 years, and two clinical nursing managers, all with postgraduate degrees or above, were engaged in evaluating and revising the Chinese version on semantics, language expression habits, and professional nature. The third version was generated after adaptation and modification according to Chinese culture and language habits.

#### Step 2: Pretest

Fifteen-five ward nurses who met the inclusion criteria were selected by convenience sampling. It is important to note that the investigators explained to the respondents the purpose and significance of the study and obtained their informed consent before sending the scales. Following that, interviewers questioned survey participants about whether the scale contained ambiguous, incomprehensible, or disagreeable items. Based on the consistent feedback from the interviews, the final Chinese version of the ATREND scale was completed after the last revision of the scale was corrected and proofread.

### Questionnaire design

#### Background characteristics

The team designed the General Demographic Characteristics Questionnaire after a comprehensive literature review. Six items were required to be self-reported by participants: age, gender, educational level, work experience, title, and department.

#### The ATREND scale

The ATREND scale is an 11-item scale developed by Chua et al. (2022) to comprehensively evaluate nurses’ attitudes toward recognizing early signs of clinical deterioration. The ATREND scale includes three dimensions: (1) beliefs about the importance of patient observation (6 items), (2) use of broader patient assessment skills (2 items), and (3) confidence in recognizing clinical deterioration (3 items). Five-point Likert scale was applied (1 = strongly disagree, 2 = disagree, 3 = neutral, 4 = agree, 5 = strongly agree). Items 7 and 8 were reversely coded, with the rest scoring positively. Those who score higher indicate a positive attitude toward recognizing clinical deterioration in its early stages. The original scale has acceptable internal consistency, with a Cronbach’s alpha of 0.745 for the overall scale and 0.637 to 0.763 for the subscales.

#### V-scale

Mok et al. (2015a) developed the V-scale administered as a comparative measure for assessing the criterion validity of the ATREND scale. Nursing attitudes toward vital sign monitoring are measured using the V-scale in identifying, interpreting, and reporting patients’ deterioration. The Chinese version of the scale, translated and localized by Zheng et al. (2019) in 2019, has been verified to have good reliability and validity. A total of 16 items are included in this scale which measures five dimensions: critical indicators of vital signs deterioration, knowledge, workload, communication, and operational skills. Important sign-monitoring attitudes were rated on a Likert scale ranging from “1 = strongly disagree” to “4 = strongly agree.” A higher total score reflects more positive attitudes toward vital signs monitoring.

### Data collection

Face-to-face distribution of questionnaires was conducted from March 2022 to July 2022 with the consent of the hospital nursing department leadership. Participants filled out the questionnaires independently in a quiet classroom arranged by nurse managers. Forty randomly selected nurses from the sample completed the questionnaires 2 weeks later to test the reliability of the questionnaire.

### Statistical analysis

The statistical analysis was performed using SPSS 25.0 (IBM Corp., Armonk, NY, United States) and AMOS 23.0 (IBM Corp., Armonk, NY, United States).

### Items analysis

The total score was ranked from highest to lowest. By an independent sample *t*-test to evaluate the discriminatory properties of the translated scale, the relationship between the high group (highest 27%) and the low group (lowest 27%) was analyzed (McCowan and McCowan, 1999). The correlations between the items, the translated scale, and Cronbach’s α coefficient of item deletion were analyzed. It was done to determine if the items of the translated scale could be retained.

### Reliability analysis

Cronbach’s coefficients, split-half reliability, and retest reliability were calculated to determine the reliability of the translated scale. A Cronbach’s coefficient of at least 0.7 was considered acceptable (Chang et al., 2018). The translated scale items were divided into two parts based on odd and even numbers, and the correlation between the results of the two sides was calculated to assess split-half reliability (Ren et al., 2021). With the translated scale, 40 nurses from different wards were retested 2 weeks later to determine the scale’s retest reliability.

### Validity analysis

Seven relevant experts were invited (2 professors from Jinzhou Medical University’s Nursing College, one associate chief nurse, two nurse practitioners with over 10 years of clinical experience, and two clinical nursing managers) to assess the content validity of the ATREND scale. These experts were selected based on: (i) their extensive expertise in clinical deterioration, RRSs, and nursing-related expertise; (ii) their familiarity with the manual steps of the scale and psychometric measures; (iii) their bachelor’s degree or higher and at least 10 years of experience in the field; (iv) their rigorous and pragmatic approach to research; and (v) their volunteers to participate in this study. Based on the content, the Likert 4-point rating system was used to collect expert responses (1 = irrelevant, 2 = weakly relevant, 3 = strongly relevant, 4 = highly relevant). Irrelevant and weakly relevant are assigned 0 points, and strongly relevant and highly relevant are assigned 1 point. In this study, both item content validity indexes (I-CVI) and scale content validity indices (S-CVI) were calculated based on the items and scales, respectively (Lynn, 1986). Exploratory factor analysis (EFA) and confirmatory factor analysis (CFA) were conducted to evaluate the underlying factor structure of the translated scale. A random sample of 460 cases was divided into two groups by SPSS software; one for EFA (*n* = 230) and one for CFA (*n* = 230). In terms of characteristics, both groups were similar. It has been suggested that the data were suitable for factor analysis based on the premise that Kaiser–Meyer–Olkin (KMO) >0.6 (Kaiser, 1970, 1974) and Bartlett’s spherical test yielded *p* < 0.05 (Bartlett, 1954). An EFA was conducted using maximum variance rotation on the scale items. Generally, contributions over 50% are considered acceptable, and contributions over 70% are deemed good (Diamond et al., 2014). The structural validity of the scale was tested using AMOS 23.0 in CFA. Several fit indexes must be good for the first-order three-factor structure of the ATREND scale to be replicable: χ^2^/DF, goodness-of-fit index (GFI), adjusted GFI (AGFI), root-mean-square error of approximation (RMSEA), Tucker–Lewis index (TLI), and comparative fit index (CFI) and incremental fit index (IFI). Generally speaking, *χ*^2^/DF values <3 indicated a good fit and RMSEA values <0.08 demonstrated good adaptability and good model fit (Bentler and Bonett, 1980; Ondé and Alvarado, 2020), while the remaining indicators values >0.9 indicated a good fit; however, a value >0.8 suggests that the model is acceptable (Hu and Bentler, 1999).

### Convergent validity

**Figure 1:**
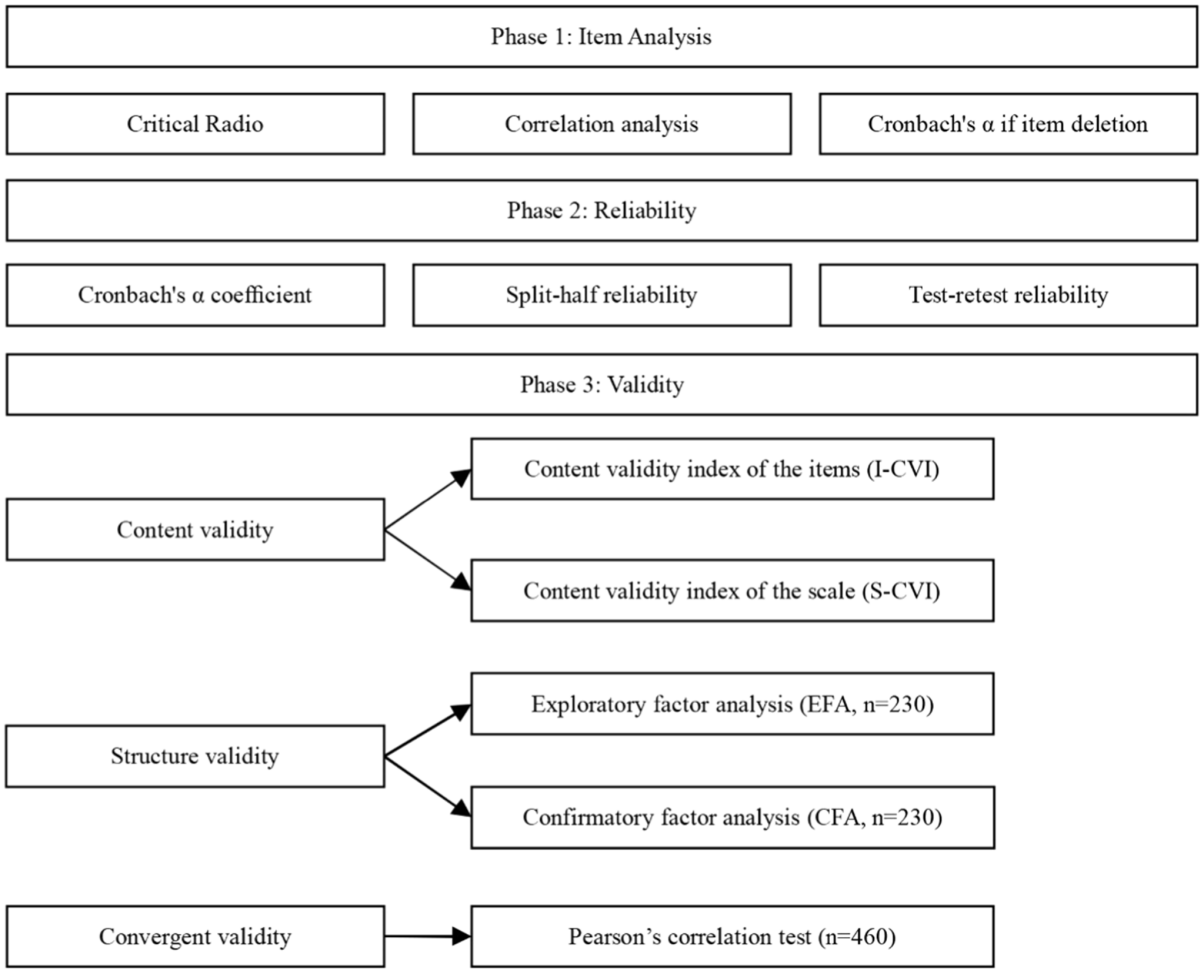
The data analysis procedure for Chinese version of the ATREND.

Calibration correlation refers to using a recognized valid scale as a standard to test the degree of correlation between the measuring and standard scales. As a comparator, the V-scale was used in this study. A correlation study was conducted between the Chinese ATREND scale and the V-scale using Pearson’s correlation analysis. In the case of *r* > 0.7, the test has a high level of validity. The test is moderately valid when 0.4 < *r* < 0.7. It has a low validity when *r* < 0.4 (Wang and Sun, 2011). Figure 1 shows the steps for the statistical analysis of the data.

## Results

### Descriptive statistics

This study included 460 ward nurses: 32 males (7.0%) and 428 females (93.0%). Participants aged 25–34 years accounted for 60.0%. Forty-five percent of the participants had an undergraduate education. The largest proportion of participants were nurse practitioners (59.1%); in terms of years of experience, 40.0% of participants had been working for 6 to 10 years. The participants included 244 internal medicine nurses (53.0%), accounting for the largest. This study meets the requirements of the Declaration of Helsinki. For the specific sociodemographic information, see Table 1.

**Table 1:** Frequency distribution of demographic characteristics (*n* = 460).

Factors	Group	*n*	%
Age	18–24	52	11.3
	25–34	276	60
	35–44	125	27.2
	≥45	7	1.5
Gender	Male	32	7.0
	Female	428	93.0
Education level	Technical secondary school education	28	6.1
	Junior college education	166	36.1
	Undergraduate education	207	45
	Postgraduate education	59	12.8
Working experience (year)	<1	19	4.1
	1–5	106	23.0
	6–10	170	40.0
	>10	165	35.9
Title	Nurse	51	11.1
	Nurse Practitioners	272	59.1
	Nurse Supervisor	133	23.9
	Associate Chief Nursing Officer	4	0.9
Departments	Internal Medicine	244	53.0
	Surgery	143	31.1
	Other (Orthopedics, obstetrics, gynecology, etc.)	73	15.9

### Item analysis

A critical ratio (CR) greater than 3.000 indicates that items are more discriminable. There was good discrimination between 11 items in the translated scale, as the CR ranged from 10.354 to 16.687. Scores for each item were positively correlated with the total score (*r* = 0.362–0.577, *p* < 0.001), showing a correlation between items and scales. After deleting each item, Cronbach’s α value of the translated scale was 0.779 to 0.800, which does not exceed Cronbach’s α value of the scale (0.804; Table 2).

**Table 2:** Item analysis for the Chinese version of the ATREND scale.

Item	Item score	Critical ratio	The correlation coefficient between the Item and the total score	Cronbach’s alpha, if an item deleted
a1	2.99 ± 0.82	16.687	0.546	0.782
a2	3.03 ± 0.83	15.943	0.577	0.779
a3	3.07 ± 0.81	13.796	0.470	0.789
a4	3.04 ± 0.84	14.983	0.525	0.783
a5	3.13 ± 0.84	15.726	0.562	0.780
a6	3.08 ± 0.85	13.218	0.493	0.786
a7	2.98 ± 1.11	10.354	0.391	0.798
a8	3.13 ± 1.14	11.223	0.397	0.799
a9	3.05 ± 0.95	11.964	0.401	0.795
a10	3.06 ± 1.03	14.325	0.489	0.786
a11	3.13 ± 0.98	11.511	0.362	0.800

### Reliability analysis

According to Cronbach’s α value, the translated scale was 0.804, and the dimensions ranged from 0.782 to 0.863. The result of this study showed a split-half reliability of 0.846. The test–retest reliability was 0.711 when 40 ward nurses were randomly selected to retest after 2 weeks (Table 3).

**Table 3:** Reliability analysis for the Chinese version of the ATREND scale.

The scale and its dimension	Score	Cronbach’s alpha	Split-half reliability	Test–retest reliability
The ATREND scale	33.70 ± 5.97	0.804	0.846	0.711
Factor 1	18.35 ± 3.84	0.863		
Factor 2	6.11 ± 2.04	0.782		
Factor 3	9.24 ± 2.55	0.831		

### Validity analysis

#### Content validity analysis

A panel of seven experts evaluated the translated scale for content validity. As shown in Table 4, the I-CVI for the translated scale was 0.857 to 1.000, and the S-CVI was 0.922.

**Table 4:** Content validity analysis for the Chinese version of the ATREND scale.

Item	Experts (score)							I-CVI
	1	2	3	4	5	6	7	
a1	1	1	1	1	1	1	1	1
a2	1	0	1	1	1	1	1	0.857
a3	1	1	1	1	1	1	1	1
a4	1	1	1	1	1	1	0	0.857
a5	1	1	1	1	1	1	1	1
a6	1	1	1	1	1	0	1	0.857
a7	1	1	1	1	1	1	1	1
a8	1	1	1	0	1	1	1	0.857
a9	1	1	1	1	1	1	0	0.857
a10	1	1	1	1	1	1	1	1
a11	0	1	1	1	1	1	1	0.857

#### EFA

**Figure 2:**
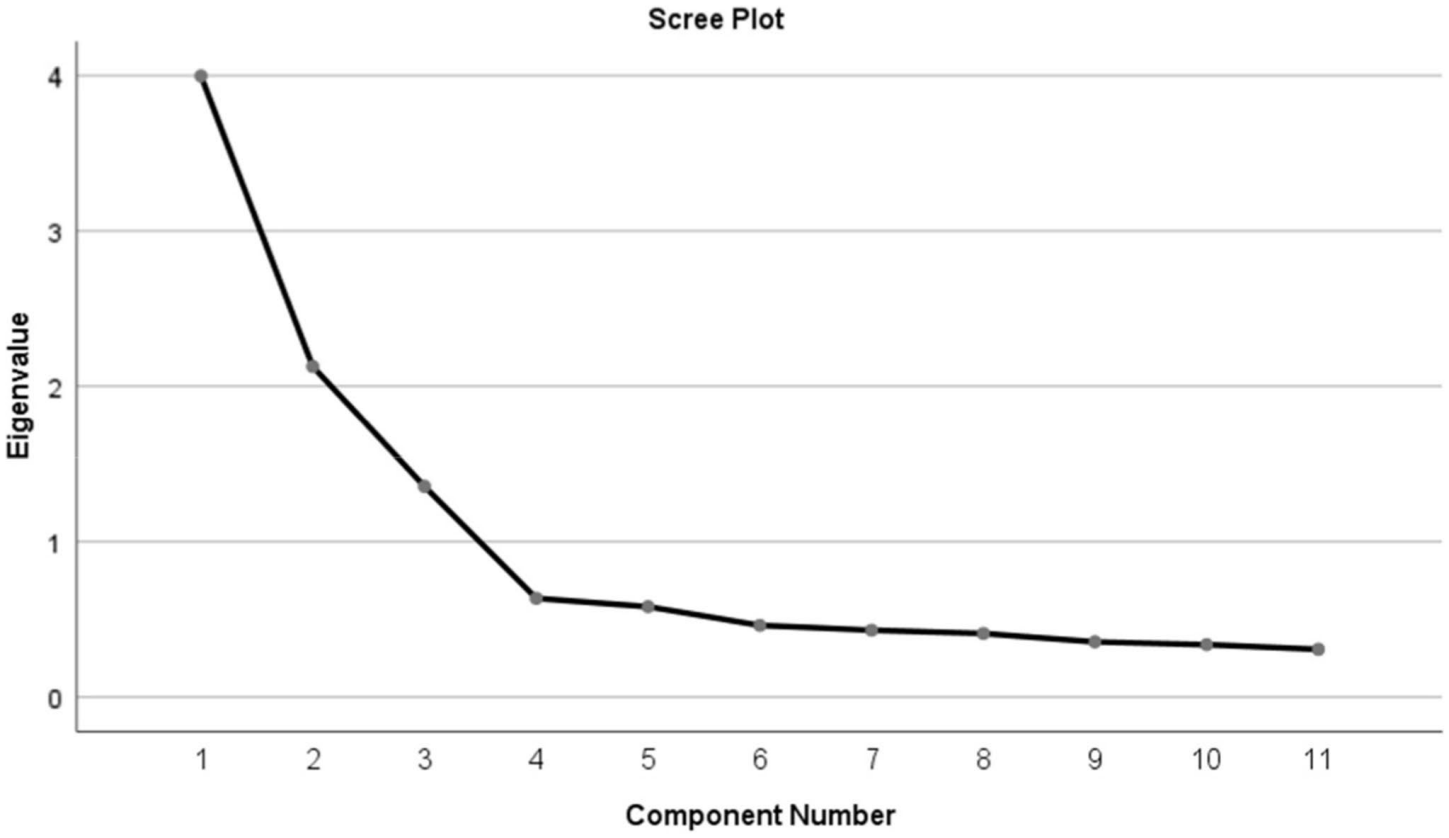
Screen plot of exploratory factor analysis for Chinese version of the ATREND.

The Kaiser–Meyer–Olkin measure of sampling adequacy was 0.807, and the Bartlett test of sphericity was significant (*χ*^2^ = 1047.815; *p* < 0.001). Thus, the matrix is not an identity matrix and can be used for factor extraction. As determined by Kaiser’s rule, three factors explained 69.166% of the variance with initial eigenvalues greater than 1. In Figure 2, the descending tendency became weaker after the third point, confirming the three-factor structure of the original scale. As a result of varimax rotation, 36.491, 19.485, and 13.191% of variance were explained by the three factors. Aside from that, the loadings of the factors are also satisfactory, as displayed in Table 5.

**Table 5:** Factor loadings of exploratory factor analysis for the Chinese version of the ATREND scale.

Item	Factor 1	Factor 2	Factor 3
a1	0.770	−0.199	−0.003
a2	0.793	−0.180	−0.101
a3	0.688	−0.287	−0.072
a4	0.754	−0.190	−0.080
a5	0.787	−0.176	−0.081
a6	0.709	−0.224	−0.152
a7	0.352	0.242	0.805
a8	0.392	0.279	0.774
a9	0.248	0.799	−0.228
a10	0.457	0.703	−0.227
a11	0.284	0.775	−0.221

#### CFA

**Figure 3:**
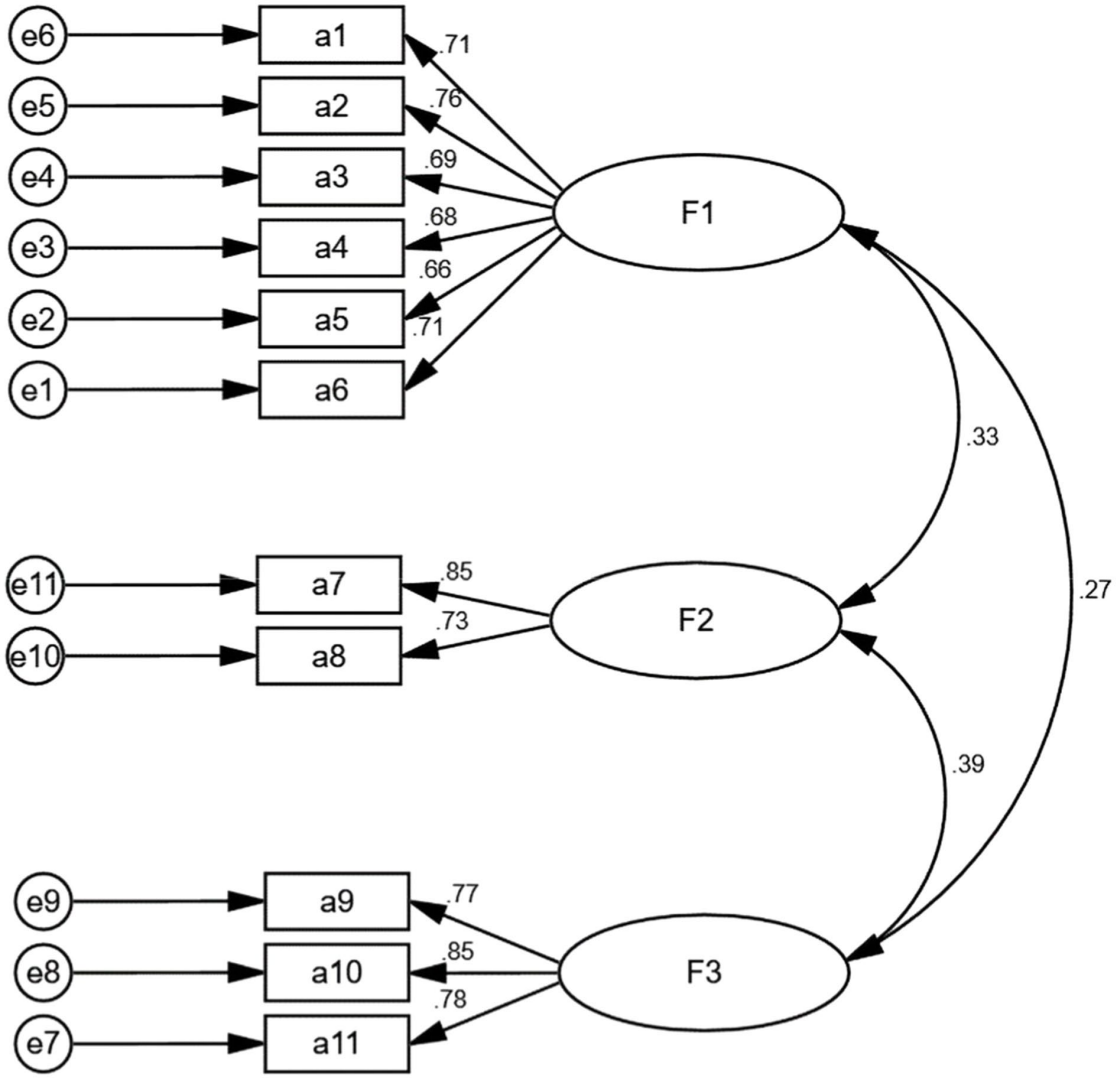
Standardized three-factor structural model of the ATREND (*n* = 230).

Figure 3 shows the results of the CFA. Based on the CFA, the model fit the data well (*χ*^2^/DF = 1.498 < 5, GFI = 0.954 > 0.9, AGFI = 0.926 > 0.9, CFI = 0.978 > 0.9, RMSEA = 0.047 < 0.08, IFI = 0.979 < 0.9, TLI = 0.971 > 0.9).

### Convergent validity

This study used the Chinese version of the V-Scale as the calibration scale to analyze its correlation with the ATREND scale dimensions and total score. The results showed a positive correlation between the two scales (*r* = 0.440, *p* < 0.01). A significant correlation coefficient was found between different dimensions and the V-Scale: 0.351, 0.258, and 0.294 (*p* < 0.01).

## Discussion

To evaluate ward nurses’ attitudes and practices regarding early recognition of clinical deterioration, we cross-culturally adapted the ATREND scale and validated it with 460 nurses in this study. For the first time, the ATREND scale has been applied to a Chinese population with good construct validity, discriminant validity, and reliability. It can be used to predict nurses’ alertness to clinical deterioration. It also benefits the development of an evidence-based training program to improve nurses’ alertness to early recognition of clinical deterioration, which is essential to ensure nursing safety and enhance satisfaction with the quality of service.

Based on the modified Brislin translation principle (Brislin, 1970; Jones et al., 2001), the ATREND scale was translated into Chinese. Seven nursing experts were invited to assess the form of the ATREND scale’s semantics, language expression habits, and professional nature, as well as the validity of its content. The experts reviewed the items for content validity and agreed that the scale showed good content validity in its original form, with all experts agreeing on the scale items.

A preliminary survey of 40 ward nurses found that the Chinese version of the ATREND scale had a clear, easy-to-understand semantic expression and a reasonable scale structure. In addition, the CR value of each project is well above 3. The scores of each item were moderately to highly correlated with the total score. The item-total correlation of item 11 was relatively low but acceptable. It is proved that this analysis has statistical significance and can be applied to the analysis of this study. It may be related to the fact that junior nurses have difficulty in detecting changes in patients’ vital signs in their clinical work and also have difficulty in connecting them to pathophysiological changes in the disease, making poor judgments about the regression and prognosis of the disease, thus relying heavily on vital sign readings and marginalizing other patient assessment skills (Keene et al., 2017; Keers et al., 2018). After deleting each item, Cronbach’s α value did not exceed the original value of the translated scale. The above indicates that the 11 items in the Chinese version of the ATREND scale can be retained with good discrimination.

The reliability of the ATREND scale’s Chinese version is comparable to the original English version (Chua et al., 2022). The reliability analysis reflects the instrument’s authenticity by measuring its consistency and stability (Koo and Li, 2016). Using internal consistency, test–retest, and split-half reliability, we evaluated the reliability of the Chinese version of the ATREND scale. The internal consistency, expressed by Cronbach’s α value, reflects the homogeneity among all items in the scale (Anselmi et al., 2019). In this study, Cronbach’s α value for the translation scale was 0.804 (English version 0.745), and for each dimension, the coefficients ranged from 0.782 to 0.863 (English version 0.637 to 0.763), which was higher than that of the original study (Chua et al., 2022). Test–retest reliability in research refers to the consistency of results obtained by repeatedly measuring the same subjects (Leppink and Pérez-Fuster, 2017). Test–retest reliability analyses rarely achieve perfect results (Wikman and Wärneryd, 1990). Our result (retest reliability = 0.711) suggests that ward nurses’ attitudes toward early recognition of clinical deterioration have reasonable temporal variability, from which it would be valuable to consider some form of an educational intervention to change ward nurses’ attitudes toward early recognition of clinical deterioration. Regarding the retest reliability of the ATREND scale, our results were better than the standard value, showing that the scale reliably measures ward nurses’ attitudes toward early recognition of clinical deterioration and that the scale has measurement stability across time. Overall, the Chinese version of the ATREND scale shows good reliability among ward nurses.

Validity refers to the degree to which a measurement instrument or tool can accurately measure the thing to be measured (Kimberlin and Winterstein, 2008). This study evaluates the validity of the Chinese version of the ATREND scale from three aspects: content validity analysis, structure validity analysis, and convergent validity analysis. A total of seven nursing experts were invited to comment on the content validity of the Chinese version for each item, and the final assessment found that the I-CVI ranged from 0.857 to 1.000; the S-CVI averaged 0.922. According to the evaluation criteria, the I-CVI must be greater than 0.78, and the S-CVI must reach 0.80 when the number of experts exceeds 5, so the scale shows good content validity. Structure validity reflects the degree of integration of a scale with the theoretical or conceptual framework on which it is based and is often measured by EFA (Kang, 2013). Good structural validity is generally defined as (1) the factors that emerged from EFA explain more than 50% of the scale and (2) each item has a high loading value (>0.4) on one common factor and a low loading value on the other common factors.

According to EFA, the three common factors extracted in this study contributed 69.166% to the cumulative variance contribution, which is higher than the original scale (56.30%), indicating that the entries had strong explanatory power for nurses’ attitudes about vital sign monitoring for identifying changes in patients’ conditions. In this study, all fitting indices met the judgment standard, suggesting that the Chinese version of the ATREND scale has a good overall fit. Meanwhile, the CFA results showed that the Chinese version of the ATREND scale’s fit indices met or exceeded the original report’s fit indices. The Chinese version of the ATREND scale has a better fit.

We also chose the V-scale to assess convergent validity. The Chinese V-scale has good validity and is widely used to assess ward nurses’ attitudes toward monitoring vital signs for identifying patient deterioration (Zheng et al., 2019). Therefore, using the V-scale to assess the convergent validity is reasonable. Pearson’s correlation analysis showed that the correlation coefficient between the two scales (*r* = 0.440, *p* < 0.001) showed a moderately significant positive correlation. Overall, the Chinese version of the ATREND scale has suitable validity among ward nurses.

## Limitation and perspectives

However, there are certain limitations of this study. First, this scale is a self-assessment scale, and the study participants tend to choose the correct answer rather than their true thoughts subconsciously, and bias is inevitable. Second, the sample was selected for convenience sampling, and the nurses in the sample were generally highly educated. Previous studies have shown that education is an influential factor in nurses’ vital sign-monitoring attitudes toward identifying patient deterioration, so bias in the sample population’s education may affect the questionnaire’s generalizability. Third, due to the limited number of male nurses working in nursing practice, the total number of female nurses in the sample of this study was much higher than that of male nurses, and future studies could recruit more male nurses to conduct related studies. Statistical testing of the scale is a long-term task, and it is suggested that further testing of the Chinese version of the ATREND scale may be conducted in the future with a balance of educational qualifications, etc.

## Conclusion

The English version of the ATREND scale has been successfully translated and culturally adapted for use in China, and its psychometric properties have been validated among ward nurses. Moreover, factor analysis has shown that the Chinese version of the ATREND scale is consistent with the original scale in terms of dimensions and is reliable and valid. In the background of the Health China strategy, this scale can effectively assess ward nurses’ attitudes toward vital sign monitoring for identifying changes in patients’ conditions, which is essential for nursing managers to organize comprehensive training programs to reduce the occurrence of adverse events and improve nursing safety and service quality.

## Data availability statement

The raw data supporting the conclusions of this article will be made available by the authors, without undue reservation.

## Ethics statement

The studies involving human participants were reviewed and approved by the Ethics Committee of the Jinzhou Medical University (no. JZMULL2022025). The participants provided their written informed consent to participate in this study.

## Author contributions

WL, HY, BL, YZ, and MF involved in the study route design and data collection. After data collection and analysis. WL wrote the draft. HY and BL made essential revisions to the draft to identify important intellectual content. YZ and MF: data collection and statistical analysis. All other co-authors also made critical contributions to the revision of the manuscript.

## Conflict of interest

The authors declare that the research was conducted in the absence of any commercial or financial relationships that could be construed as a potential conflict of interest.

## Publisher’s note

All claims expressed in this article are solely those of the authors and do not necessarily represent those of their affiliated organizations, or those of the publisher, the editors and the reviewers. Any product that may be evaluated in this article, or claim that may be made by its manufacturer, is not guaranteed or endorsed by the publisher.
