# Supplementary material for: The transcultural adaptation and validation of the Chinese version of the Attitudes Toward Recognizing Early and Noticeable Deterioration scale
Source: Front Psychol. 2022 Dec 6;13:1062949. doi: 10.3389/fpsyg.2022.1062949 (PMC9765647; doi:10.3389/fpsyg.2022.1062949)
Supplement: Supplementary file 1 [file Table_1.docx]

**Supplementary Table 1.** The comparison between English and Chinese item.

| No. | English item | Chinese item |
| --- | --- | --- |
| 1 | I believe that most cardiopulmonary resuscitation events can be avoided with recognition and response to early signs of clinical deterioration. | 我认为通过识别和应对临床恶化的早期迹象可以避免大多数心肺复苏事件。 |
| 2 | I believe that most episodes of clinical deterioration are detected when the patients develop early signs and symptoms of deterioration. | 我认为大多数临床恶化的发作都是在患者出现早期体征和症状恶化时被检测到的。 |
| 3 | I tend to recognise signs of clinical deterioration through both vital signs assessment and other patient assessment. | 我倾向于通过评估生命体征评估和其他患者评估项来识别临床恶化的迹象。 |
| 4 | I believe ongoing patient assessment that goes beyond vital signs monitoring is necessary to detect early signs of clinical deterioration. | 我认为，为了发现临床恶化的早期迹象对患者进行除了生命体征监测之外的持续评估是有必要的。 |
| 5 | When I have delegated the task of vital signs monitoring to other nursing staff, I check over the recorded vital signs to ensure that there are no abnormalities that have been missed or not reported to me. | 当我将生命体征监测任务委托给其他护理人员时，我会检查生命体征记录表，以确保没有遗漏或未报告给我的异常情况。 |
| 6 | I believe that nursing staff delegated to the task of vital signs monitoring are responsible for reporting any abnormality to me. | 我认为负责监测生命体征的护理人员有责任向我报告任何异常情况。 |
| 7 | Other than vital signs assessment, I rarely perform other patient assessment to assess for early signs of clinical deterioration. | 除了生命体征评估，我很少进行检测临床恶化早期迹象的其他患者评估。 |
| 8 | Other than vital signs assessment, I do not see the need to perform other patient assessment to detect early signs of clinical deterioration. | 除生命体征评估外，我认为不需要进行其他患者评估来检测临床恶化的早期迹象。 |
| 9 | I am confident in recognising early signs of clinical deterioration. | 我有把握发现临床恶化的早期迹象。 |
| 10 | I am confident in performing patient assessment using a structured approach (e.g., ABCDE approach: Airway, Breathing, Circulation, Disability, Expose) to assess for clinical deterioration. | 我有把握使用结构化方法（如ABCDE方法：气道、呼吸、循环、残疾、暴露）对患者进行临床恶化评估。 |
| 11 | I lack confidence in recognising early signs of clinical deterioration that may not be reflected in a patient's vital signs. | 我没有把握能识别临床恶化的早期迹象，这些迹象可能无法通过患者的生命体征反映出来。 |

*Note: The original questionnaire of the Chinese ATREND includes 11 items distributed in beliefs about importance of patient observation (Items 1-6), use of broader patient assessment skills (Items 7-8), confidence in recognising clinical deterioration (Items 9-11).*
