# Supplementary material for: The transcultural adaptation and validation of the Chinese version of the Attitudes Toward Recognizing Early and Noticeable Deterioration scale
Source: Front Psychol. 2022 Dec 6;13:1062949. doi: 10.3389/fpsyg.2022.1062949 (PMC9765647; doi:10.3389/fpsyg.2022.1062949)
Supplement: Supplementary file 2 [file Table_2.docx]

**Supplementary Table 2.** The final Chinese version of the ATREND

这份问卷评估您对您所在医院的普通病房中识别临床恶化病人的早期症状的意见和态度。请选择最符合您情况的答案。没有正确或错误的答案。

| 序号 | 内容 | 选项 | | | | |
| --- | --- | --- | --- | --- | --- | --- |
| 1 | 我认为通过识别和应对临床恶化的早期迹象可以避免大多数心肺复苏事件。 | 非常不同意 | 不同意 | 中立 | 同意 | 非常同意 |
| 2 | 我认为大多数临床恶化的发作都是在患者出现早期体征和症状恶化时被检测到的。 | 非常不同意 | 不同意 | 中立 | 同意 | 非常同意 |
| 3 | 我倾向于通过评估生命体征评估和其他患者评估项来识别临床恶化的迹象。 | 非常不同意 | 不同意 | 中立 | 同意 | 非常同意 |
| 4 | 我认为，为了发现临床恶化的早期迹象对患者进行除了生命体征监测之外的持续评估是有必要的。 | 非常不同意 | 不同意 | 中立 | 同意 | 非常同意 |
| 5 | 当我将生命体征监测任务委托给其他护理人员时，我会检查生命体征记录表，以确保没有遗漏或未报告给我的异常情况。 | 非常不同意 | 不同意 | 中立 | 同意 | 非常同意 |
| 6 | 我认为负责监测生命体征的护理人员有责任向我报告任何异常情况。 | 非常不同意 | 不同意 | 中立 | 同意 | 非常同意 |
| 7 | 除了生命体征评估，我很少进行检测临床恶化早期迹象的其他患者评估。 | 非常不同意 | 不同意 | 中立 | 同意 | 非常同意 |
| 8 | 除生命体征评估外，我认为不需要进行其他患者评估来检测临床恶化的早期迹象。 | 非常不同意 | 不同意 | 中立 | 同意 | 非常同意 |
| 9 | 我有把握发现临床恶化的早期迹象。 | 非常不同意 | 不同意 | 中立 | 同意 | 非常同意 |
| 10 | 我有把握使用结构化方法（如ABCDE方法：气道、呼吸、循环、残疾、暴露）对患者进行临床恶化评估。 | 非常不同意 | 不同意 | 中立 | 同意 | 非常同意 |
| 11 | 我没有把握能识别临床恶化的早期迹象，这些迹象可能无法通过患者的生命体征反映出来。 | 非常不同意 | 不同意 | 中立 | 同意 | 非常同意 |

Adapted with permission from Wei Ling Chua BSc (Nursing) (Hons), PhD, RN / Chua WL, Smith D, Wee LC, Ting KC, Yeo MLK, Mordiffi SZ, Liaw SY. Development and psychometric evaluation of the Attitudes Towards Recognising Early and Noticeable Deterioration (ATREND) scale. J Clin Nurs. 2022 May 8. doi: 10.1111/jocn.16350. Epub ahead of print. PMID: 35527356. / https://onlinelibrary.wiley.com/doi/10.1111/jocn.16350
